# Neural Basis of Stimulus-Angle-Dependent Motor Control of Wind-Elicited Walking Behavior in the Cricket *Gryllus bimaculatus*

**DOI:** 10.1371/journal.pone.0080184

**Published:** 2013-11-14

**Authors:** Momoko Oe, Hiroto Ogawa

**Affiliations:** 1 Graduate School of Life Science, Hokkaido University, Sapporo, Japan; 2 Department of Biological Sciences, Faculty of Science, Hokkaido University, Sapporo, Japan; 3 PREST, Japan Science and Technology Agency (JST), Kawaguchi, Japan; Claremont Colleges, United States of America

## Abstract

Crickets exhibit oriented walking behavior in response to air-current stimuli. Because crickets move in the opposite direction from the stimulus source, this behavior is considered to represent ‘escape behavior’ from an approaching predator. However, details of the stimulus-angle-dependent control of locomotion during the immediate phase, and the neural basis underlying the directional motor control of this behavior remain unclear. In this study, we used a spherical-treadmill system to measure locomotory parameters including trajectory, turn angle and velocity during the immediate phase of responses to air-puff stimuli applied from various angles. Both walking direction and turn angle were correlated with stimulus angle, but their relationships followed different rules. A shorter stimulus also induced directionally-controlled walking, but reduced the yaw rotation in stimulus-angle-dependent turning. These results suggest that neural control of the turn angle requires different sensory information than that required for oriented walking. Hemi-severance of the ventral nerve cords containing descending axons from the cephalic to the prothoracic ganglion abolished stimulus-angle-dependent control, indicating that this control required descending signals from the brain. Furthermore, we selectively ablated identified ascending giant interneurons (GIs) *in vivo* to examine their functional roles in wind-elicited walking. Ablation of GI8-1 diminished control of the turn angle and decreased walking distance in the initial response. Meanwhile, GI9-1b ablation had no discernible effect on stimulus-angle-dependent control or walking distance, but delayed the reaction time. These results suggest that the ascending signals conveyed by GI8-1 are required for turn-angle control and maintenance of walking behavior, and that GI9-1b is responsible for rapid initiation of walking. It is possible that individual types of GIs separately supply the sensory signals required to control wind-elicited walking.

## Introduction

Animals plan, select, direct and modulate their behaviors according to their environment. The neural processes underlying these adaptive behaviors require animals to perceive various types of stimuli, such as visual, auditory, chemical and mechanical stimuli, integrate these sensory inputs, to extract useful information from them, and to derive the appropriate motor outputs. The neural processing that connects spatial perception of the stimulus to stimulus-dependent control of the motor outputs is fundamental, especially in the case of directed behaviors. Sensorimotor pathways have therefore been investigated in various kinds of directed behaviors ranging from stochastic control of oriented locomotion in nematodes to complex goal-directed control of reaching in primates.

The escape response represents a useful model system for understanding the neural basis of directed behaviors [1]–[3]. The neural mechanisms responsible for the escape response, mediated by identified giant fibers, have been particularly well-studied, because the use of identified neurons enables the neuronal components and circuits underlying individual behaviors to be clarified [4]–[6]. The neural mechanism of the tail-flip escape response in crayfish has been well studied [5]. Two pairs of identified giant fibers mediate the tail-flip responses. These include lateral giants that are activated by a sudden mechanical stimulus to the posterior, such as a sharp tap to the abdomen, and evoke a lateral giant forward flip to escape from an approaching predator. The others are medial giants, which are activated by stimuli applied to the head, and evoke a medial giant backward flip. Giant fiber systems produce stereotypical actions with very short latency [5], [7]. Mauthner cells are other neurons known to trigger escape behaviors in many anamniotes, such as lampreys, sharks, teleost fishes and amphibian larvae [6]. Throughout these species, Mauthner cells are identified as a pair of interneurons at a specific location in the brain stem. Each Mauthner neuron is activated by an auditory stimulus to the ipsilateral side to its soma and triggers a fast escape response termed ‘C-start’. The lateral and medial giants in crayfish and Mauthner cells in fishes and amphibian larvae mediate powerful and simple escape behaviors, but are not involved in the refined control of the direction of locomotion in response to the orientation or location of the stimulus source.

Wind-elicited behavior is a typical escape response in insects such as cockroaches, locusts and crickets. It is generally mediated by the cercal mechanosensory system. In this system, air-current stimuli are received by the cerci, which are two antenna-like appendages at the rear of the abdomen, and evoke various types of escape behaviors, such as walking and jumping [8]–[13], unlike the Mauthner system, which only triggers stereotypical swimming, C-start. The neural mechanism of the cercal sensory system has been studied in the crickets *Gryllus bimaculatus* and *Acheta domestica*. An air current is detected by approximately 1,000 mechanosensory hairs on the cerci [14]–[16]. These receptor afferents project in an orderly manner in the terminal abdominal ganglion (TAG) [17]–[21] where they make synaptic contacts with several interneurons, including giant interneurons (GIs), which are identified as between eight and twelve types of projection interneurons. GIs display distinct direction-specific sensitivities [22]–[26] and extend their axons to the thoracic and cephalic ganglia [27]. The facts that the response threshold of these GIs is similar to that of thoracic motor neurons [28] and that depolarizing current injection to a specific type of GI can modify cricket walking [27], [29] suggest a contribution of GIs to wind-elicited walking behavior. However, the functional role of each type of GI and the neural processing mediated by GIs within the cephalic and thoracic ganglia are unknown.

To describe a neural architecture for GI-mediated oriented locomotion, we focused on stimulus-angle-dependent control of wind-elicited walking behavior in the cricket *G. bimaculatus*. A short puff of air applied to the cerci elicits a short running response in the cricket [30]. In light of the direction specificity of GIs, we examined the relationships between various locomotory parameters and the angle of air-current stimulus relative to the cricket in wind-elicited walking. We developed a spherical-treadmill system, previously used to study walking behavior [30]–[32], to allow high-speed measurement of cricket locomotion. This system that implements a pair of optical mice to detect three rotation vectors of the ball's movement [33], enables simultaneous monitoring of both walking velocity on the x-y plane and turning velocity in a horizontal direction, and provides information on walking trajectory and time-course changes in the body-axis angle. Furthermore, the temporal resolution of this system is adequate for monitoring the initial reaction elicited by the air-current stimulus. We could therefore analyze the earliest phase of the walking behavior response, when it is less affected by sensory feedback caused by the walking itself. Using this treadmill system, we analyzed walking direction and change in body axis during the initial wind-elicited walking reaction, and detected dependency of these parameters on the stimulus angle, which characterizes sensory input-dependent control of locomotion in escape behavior. To clarify the neural pathway responsible for this directional control, we partly ablated the ventral nerve cords containing axonal fibers of ascending- and/or descending interneurons, and examined the effects of these ablations on walking parameters. We also used the laser-ablation technique to selectively inactivate GIs, and thus demonstrate the functional role of individual GIs in wind-elicited behavior.

## Materials and Methods

### Animals

Laboratory-bred adult male crickets (*G. bimaculatus*) (23–31 mm body length, 0.50–1.20 g body weight) were used throughout the experiments. They were reared under 12–12 hr light/dark conditions at a constant temperature of 27°C. All behavioral experiments were conducted during the light phase at room temperature.

### Treadmill system

**Figure 1:**
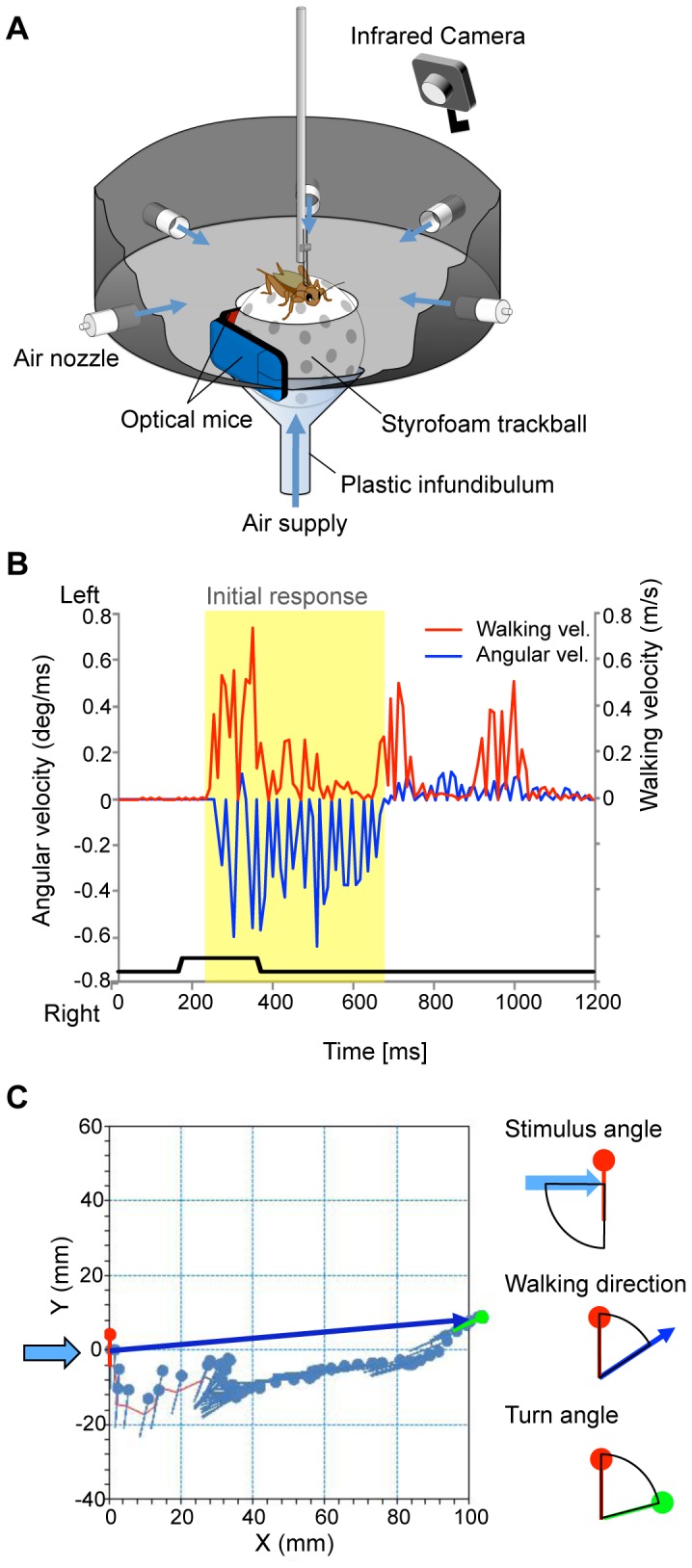
High-speed monitoring of wind-elicited locomotion using a spherical-treadmill system. A, Overview of spherical-treadmill system. B, Typical time courses of walking velocity (red trace) and turn angular velocity (blue trace) in response to air-current stimulus applied from left side (−90° stimulus angle). Bottom trace represents air-current stimulus. C, Virtual walking trajectory and body axis during the initial response represented by yellow shaded region in B. Each mark represents a cricket's location monitored at 10-ms intervals. Body axis is indicated by a line and head direction by a circle. Right diagrams represent the stimulus angle (upper), walking direction (middle) and turn angle (lower). The stimulus angle was defined as the angle between the air-flow direction (light blue arrow) and the cricket's body axis at the starting point (red line). The walking direction was measured as the angle between the body axis at starting (red line) and the line connecting the start and finish points of the initial response (blue arrow). The turn angle was measured as the angle made by the body axes at the start (red line) and finish points (green line).

We developed a spherical-treadmill system to monitor the cricket's locomotion at high temporal resolution (Figure 1A). The animal was tethered on top of a styrofoam ball (ø = 60 mm, 2.3 g) using a pair of insect pins bent into an L-shape that were stuck to cricket's tergite at a right angle to the animal's body axis with paraffin wax. The ball was set at the center of a circular experimental arena 24 cm in diameter and floated on an air stream. The cricket's walking was monitored as rotation of the ball at a 100- or 1000-Hz sampling rate, using two optical mice mounted orthogonally at either side of the ball. Special package software (TrackTaro, Chinou Jouhou Shisutemu, Kyoto, Japan) was used to calculate locomotory parameters such as walking velocity, distance, and direction, based on the measured ball rotation. The cricket's behavior was also monitored using an infrared CCD video camera at 30 frames/s (CAM 130 Night Vision, Timely, Tokyo, Japan). Movement of the cricket's leg and detectable rotation of the ball were considered as available walking responses to the stimulus.

### Air-current stimulation

An air-puff stimulus was provided to a stationary cricket by a short puff of nitrogen gas from a plastic nozzle (diameter 15 mm) connected to a pneumatic picopump (PV820, World Precision Instruments, Sarasota FL, USA). The velocity of the air current measured at the center of the arena was about 1.0 m/s, and the stimulus durations were regulated to 50, 100 and 200 ms. Eight nozzles for stimulation were arranged around the animal on the same horizontal plane on the inside wall of the arena. The nozzle ends were positioned at 45° angles between each other and at a distance of 75 mm from the animal. A single air-puff stimulus was delivered randomly with the direction varying across trials, and the interval of each trial was >3 min. All experiments were performed in a dark, soundproof chamber.

### Behavioral analysis

The treadmill system enabled us to monitor the horizontal turn velocity, defined as the ‘angular velocity’, and the translational velocity on the x-y plane, defined as the ‘walking velocity’. When crickets received an air-puff stimulus, they exhibited a few intermittent trots followed by a brief stationary moment [30]. We defined the initial continuous trot as the ‘initial response’, which finished within several hundred milliseconds (Figure 1B). The duration of the initial response was the period between the onset and termination of the first trot. The termination of the initial response was defined as the point at which both angular and walking velocities became zero, following their local maxima. ‘Walking direction’ and ‘turn angle’ were measured at the termination point of the initial response. As illustrated in Figure 1C, the walking direction was measured as the angle between the cricket's body axis at response onset and a line connecting the start and finish points of the initial response. Likewise, the turn angle was measured as the angle made by the body axes at the start and finish points. To analyze the relationships between the stimulus angle and walking direction or turn angle, we arranged the stimulus angle for backwards as 0°, clockwise as minus, and counterclockwise as plus, and arranged the walking direction and turn angle for forward as 0°. The trajectory length of the initial response was calculated and referred to as the ‘walking distance’. We also measured the response latency as the time delay from onset of the air-current stimulus to onset of the initial response.

### Free-moving experiments

We also examined the walking direction and turn angle in free-moving animals to confirm that tethering did not affect their response. The center hole for the treadmill in the floor of the experimental arena was covered with cardboard (ø = 220 mm). Crickets were able to walk freely on this board instead of on the floating ball. A 50-mm diameter circle was painted at the center of the cardboard, and the cricket was placed in it under an inverted beaker (ø = 50 mm). The beaker was carefully lifted up before applying the air-puff stimulus. The cricket's movement was monitored using an infrared camera, and the initial response was estimated from video images. The walking direction and turn angle were measured from the video images using commercial drawing software (Canvas X ACD systems, Miami, FL, USA). Walking direction was measured by drawing a line connecting the intersections between the middle legs and the animal's midline at the start and finish points, respectively.

### Ventral-nerve-cord ablation

We ablated the ventral nerve cords at various locations to examine the roles of the ascending and descending pathways. One (hemi-) or both (ambi-) sides of the nerve cords were cut between the subesophageal (SOG) and prothoracic ganglia (PTG), or between the 4th abdominal (4th AG) and TAG. Crickets were anesthetized with ice and fixed on a silicon platform, ventral side up. The epidermal membrane of the ventral surface at the cricket's neck was incised to expose the nerve cords between the SOG and PTG. The targeted nerve cords were then cut using micro-scissors. To ablate the nerve cords between the 4th AG and TAG, a small piece of the abdominal sternite was removed to expose the nerve cords, which were then cut anterior to the TAG, and the piece of sternite cuticle was replaced on the incision. Sham, control operations followed the same procedure, including anesthetic treatment and incision, but without cutting the nerve cord. Treated crickets were left to recover for 1–2 hr with sufficient water and food at room temperature before behavioral analysis.

### Laser ablation of single neurons and electrophysiology

We selectively inactivated single GIs by photo-ablation. Anesthetized crickets were mounted on a silicon platform ventral side up, and the TAG was exposed by dissection and subjected to nerve-cord ablation. The effect of anesthesia was prolonged by covering the cricket's head and thorax with a refrigerant pack. The TAG was held up using a stainless steel spoon to maintain mechanical stability during intracellular recording. Thin-wall capillaries (ø = 1.0 mm, World Precision Instruments, Sarasota, FL, USA) were pulled on a micropipette puller (PN-30, Narisige, Tokyo, Japan) to produce glass electrodes. The tip of the electrode was filled with saturated 6-carboxyfluorescein (λ_ex_ = 492 nm) in 150 mM potassium acetate. The electrode resistance varied from 20–60 MΩ. The recorded neuron was defined as a candidate GI if action potentials were elicited by air-current stimulation (velocity: 2.6 m/s, duration: 100 ms) to the cerci. 6-carboxyfluorescein was injected iontophoretically into the GI using a hyperpolarizing current (−3 nA) for 2–5 min. An argon ion laser (488 nm, 25 mW) was used for irradiation of the GI. The beam was focused through an objective lens (Plane-NEOFLUAR 10×/0.30 N.A., Carl Zeiss), and delivered to the GI axon using a confocal laser scanning microscope (Micro Radiance, BioRad Laboratories, Hercules, CA, USA). The cell-ablated cricket was left to recover for 3–4 hr at room temperature before behavioral analysis, with sufficient food and water. Sham-operated controls were subjected to the same procedures as ablated animals, including laser irradiation and penetration of a grass microelectrode, but without dye-loading.

The cell lethality of the laser ablation procedure was tested using intracellular recording in the isolated abdominal preparation during irradiation. After removal of the head and thorax, an incision was made along the dorsal midline of the abdomen and the gut, internal reproductive organs and surrounding fat were removed. The dissected abdominal body wall was split ventral side up and fixed with insect pins on a silicon platform, and a small piece of ventral cuticle was removed to expose the TAG. The preparation platform was held on the stage of the confocal laser microscope. After injection of 6-carboxyfluorescein through the intracellular electrode, action potentials elicited by electrical stimulation were monitored. The stimulus pulse (amplitude: 2.0–6.0 V, pulse duration: 100 µs) was applied at 1 Hz to cercal sensory nerves by two pairs of tungsten-wire hook electrodes (ø = 0.1 mm).

### Statistical analysis

Data were analyzed using a generalized linear model (GLM) and non-linear least squares model using R programming software (ver. 2.13.1, R Development Core Team). The best model was selected using Akaike's Information Criterion (AIC) [34]. AIC can be expressed as follows: 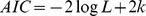
where *L* is the maximum log-likelihood and *k* is the number of parameters involved in the model. AIC is a generally-used criterion for selecting among multiple statistical models. If several models give similar maximum log-likelihood values, AIC suggests that we should select the model with the minimum number of parameters. We can therefore determine the best model with the smallest AIC among multiple models. Calculation of the AIC value was performed using the ‘glm’ or ‘AIC’ function of the R package.

To test the dependency of walking direction on stimulus angle, we first approximated the walking direction as follows:
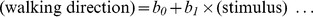
(1)


The explanatory variable in this model was stimulus (stimulus angle). *b_0_* and *b_1_* are parameters of linear predictors to be estimated. We set the probability distribution as ‘gaussian’ and link function as ‘identity’ using the R function ‘glm’. We assumed two models: model (1)-I contained the effect of stimulus angle, such that *b_0_*≠0 and *b_1_*≠0 were both estimated, while model (1)-II did not include the effect of stimulus angle, such that *b_0_*≠0 was estimated and *b_1_* = 0. We then compared AICs between models (1)-I and (1)-II. If model (1)-I was selected, walking direction was considered to be linearly correlated with stimulus angle, and if model (1)-II was selected, then walking direction was considered to be constant irrespective of the stimulus angle.

To assess the effects of experimental procedures such as changes in stimulus duration and ablation of the nerve cords or single GI, we approximated the walking direction as follows:
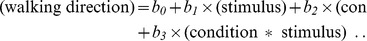
(2)where condition denotes the difference in experimental procedures. * represents the interaction of each explanatory variable. We set the probability distribution as ‘gaussian’ and link function as ‘identity’ using the R function ‘glm’. We assumed two models: model (2)-I contained the effects of stimulus angle, condition and interaction between condition and stimulus angle, such that *b_0_*≠0, *b_1_*≠0, *b_2_*≠0 and *b_3_*≠0 were all estimated, while model (2)-II did not contain the effect of condition, such that *b_0_*≠0 and *b_1_*≠0 were both estimated, while *b_2_* = 0 and *b_3_* = 0. ‘Condition’ could affect the intercept of the estimated expression, while ‘interaction’ between the walking direction and the stimulus angle could affect the slope (Figure S1). We then compared AICs between models (2)-I and (2)-II. If model (2)-I was selected, the experimental procedure affected the dependency of the walking direction on the stimulus angle.

To analyze dependency of the turn angle on the stimulus angle, we approximated the turn angle as follows, using a non-linear least squares model: 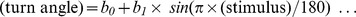
(3)


The explanatory variable in this model was stimulus (stimulus angle). *b_0_* and *b_1_* were parameters of linear predictors to be estimated. To test the effect of the stimulus angle, we assumed two models: model (3)-I contained the effect of stimulus angle, such that *b_0_*≠0 and *b_1_*≠0 were both estimated, while model (3)-II did not contain the effect of stimulus angle, such that *b_0_*≠0 was estimated and *b_1_* = 0. If the AIC of model (3)-I was smaller than that of model (3)-II, the turn angle depended on the stimulus angle in sine function. To test the effect of experimental procedures, we compared two different AICs: one AIC was the summed AICs of models for each condition, and the other AIC was for a model of combined data in both conditions. If the former AIC was selected, the experimental procedure was considered to affect the dependency of the turn angle on the stimulus angle.

Walking distance of the initial response was approximated by the following function using GLM to assess the effects of the experimental procedure: 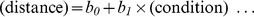
(4)


The probability distribution was set as ‘gamma’ and link function as ‘logarithm’ using the R function ‘glm’. *b_0_* and *b_1_* were parameters of linear predictors to be estimated. We assumed two models: model (4)-I contained the effect of condition, such that *b_0_*≠0 and *b_1_*≠0 were both estimated, while model (4)-II did not contain the effect of condition, such that *b_0_*≠0 was estimated and *b_1_ = 0*. We then compared AICs between models (4)-I and (4)-II. If model (4)-I was selected, the experimental procedure affected the walking distance. We analyzed the effect on response latency in the same way.

‘Response probability’ was approximated by the following expression using GLM with each stimulus angle. Each trial in which the animal exhibited wind-elicited walking behavior was assumed as 1, and those in which no such behavior was exhibited as 0. 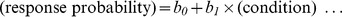
(5)


We set the probability distribution as ‘binomial’ and link function as ‘logistic’. We assumed two models: model (5)-I contained the effect of condition, such that *b_0_*≠0 and *b_1_*≠0 were both estimated, while model (5)-II did not contain the effect of condition, such that *b_0_*≠0 was estimated and *b_1_* = 0. We then compared AICs between models (5)-I and (5)-II. Selection of model (5)-I suggested that the experimental procedure affected the response probability.

The results of all the above models are listed in the supplementary tables (Table S1–S5).

## Results

### Dependency of walking direction and turn angle on stimulus angle

**Figure 2:**
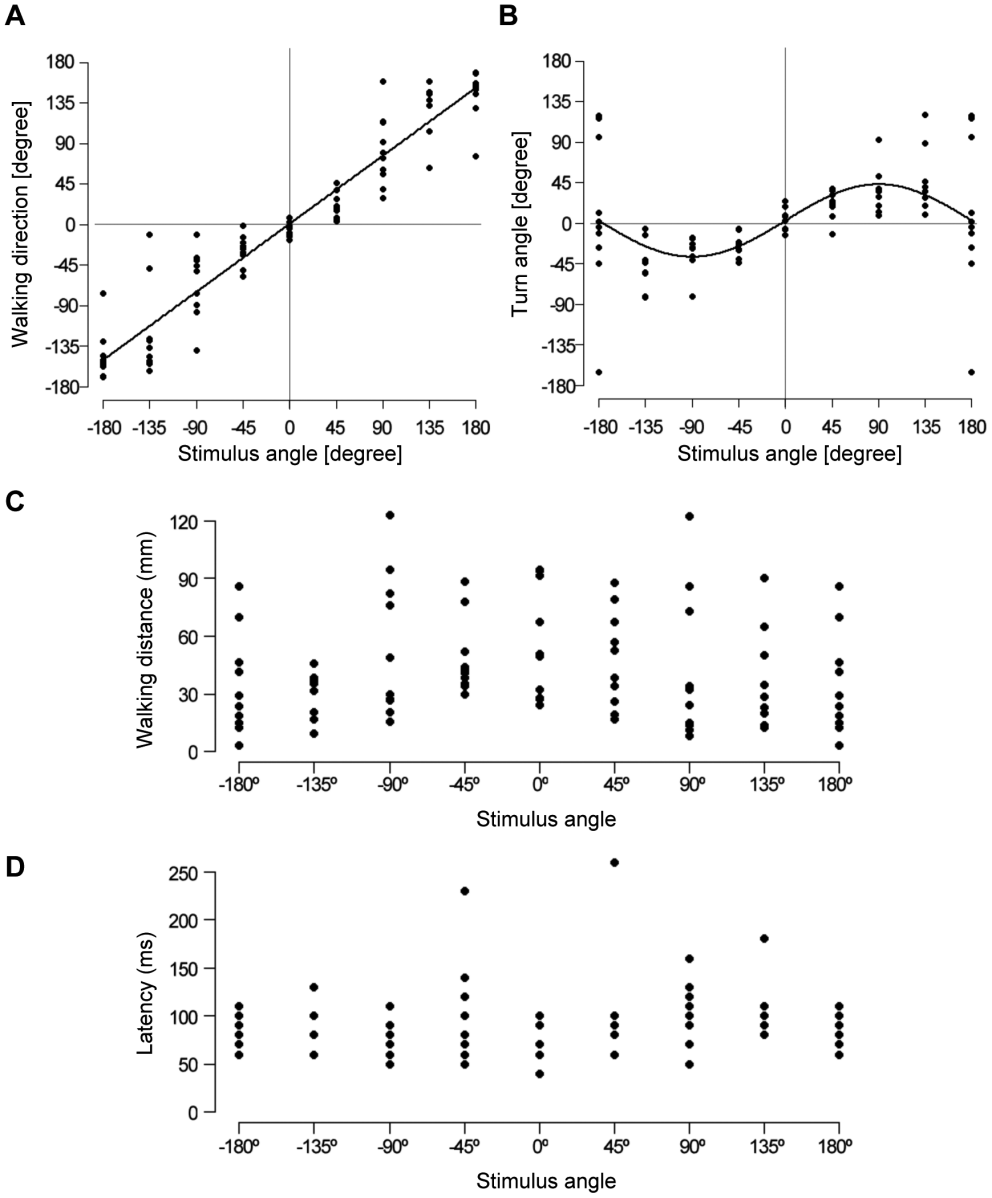
Stimulus-angle dependencies of locomotory parameters in the initial response. Each dot represents the results of a trial in an individual animal (N = 10). A, Plot of the walking direction against stimulus angle. The approximated line was expressed as 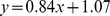. B, Plot of turn angle against stimulus angle approximated by 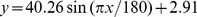. C, Plot of walking distance against stimulus angle. D, Plot of response latency against stimulus angle.

Analysis of the trajectory revealed that the cricket walked away from an air-puff stimulus and turned its head to the walking direction in initial response to a stimulus applied from the left side (−90°) (Figure 1C). As shown in Figure 1B, the walking velocity and turn-angular velocity fluctuated simultaneously. This result demonstrated that the cricket controlled both of these locomotory parameters simultaneously, even during the initial response. Based on these data, we analyzed stimulus-angle-dependent control in the initial wind-elicited walking-behavior response using the treadmill system (N = 10). Plots of the walking direction versus the stimulus angle indicated a linear relationship approximated by a positive-sloped line, which was close to the 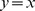 line (Figure 2A), indicating that crickets walk in the opposite direction to the air-current stimulus with precision. The walking direction depended linearly on the stimulus angle (Table S1). Plots of the turn angle versus the stimulus angle, however, indicated a different distribution approximated by a sine function (Figure 2B). The turn angle showed maximum responses to stimuli from 90° and −90° directions, such that crickets rotated their body axes maximally against air currents applied from the lateral side. The turn angle depended on the stimulus angle, according to a sine function (Table S1). These correlations between walking direction or turn angle and stimulus angle were also observed in free-moving crickets, except for a difference in the maximum amplitude of the turn angle (Figure S2). Other locomotory parameters, such as walking distance and response latency, showed no obvious correlation with stimulus angle, though the walking distance was slightly dependent on stimulus angle (Figure 2C,D). We therefore averaged these parameters regardless of stimulus angle in subsequent analyses.

### Effects of stimulus duration on directional control

**Figure 3:**
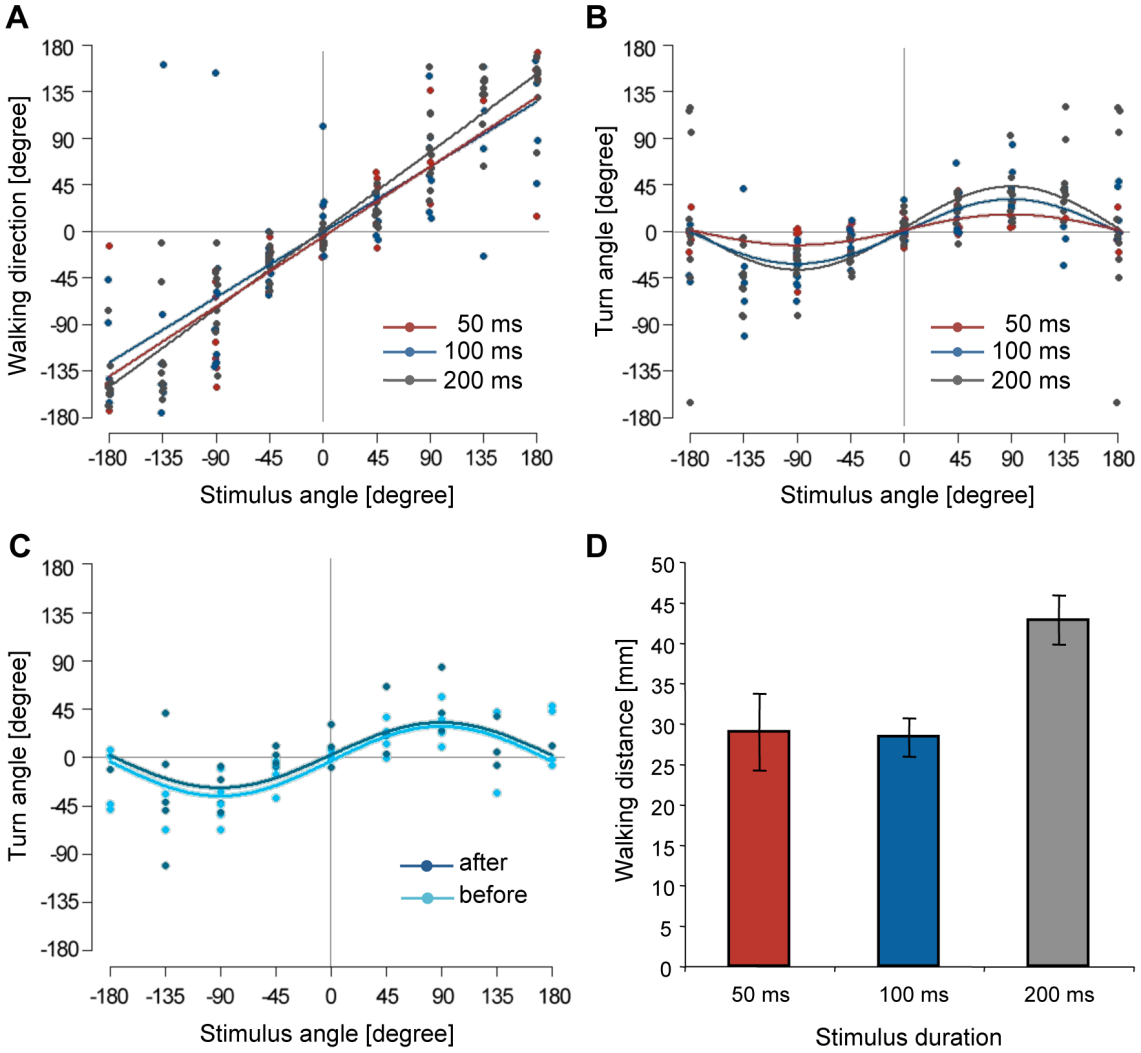
Stimulus-angle dependencies of locomotory parameters in initial responses to stimuli of various durations. The colors of dots and lines represent three different durations: gray = 200 ms (N = 10, same data set shown in Figure 2), blue = 100 ms (N = 10) and red = 50 ms (N = 10). A, Plots of walking direction against stimulus angle. The approximate lines for responses to stimuli of different durations were expressed as 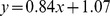 (200 ms), 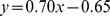 (100 ms), and 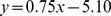 (50 ms), respectively. B, Distributions of turn angles in each response to different durations were approximated by 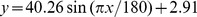 (200 ms), 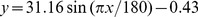 (100 ms), and 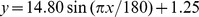 (50 ms). C, Stimulus-angle dependency of turn angle in different cases of initial responses to stimulus of 100 ms duration. Dark blue plots represent data from trials in which crickets started walking *after* the stimulus terminated. Light blue plots represent data from trials in which crickets started walking *before* the stimulus terminated. The former was approximated by 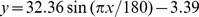, and the latter was expressed as 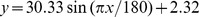. D, Walking distances during the initial responses to air-current stimuli of various durations. Columns and error bars indicate mean ± S.E.M. of all values for each stimulus duration.

The mean latency measured with the treadmill system was 86.23 ms, which include mechanical delay from the time the air puff was initiated with a picopump to the time air disturbance arrived at cerci. The actual response latency, therefore, will be 70–75 ms because the mechanical delay was approximately 10–15 ms. The result indicated that most crickets started walking before termination of the air-current stimulus, which lasted for 200 ms. We investigated the ability of a cricket to control its walking direction and turn angle in response to a stimulus that was shorter than the mean response latency. To examine the effect of stimulus duration on directional control, we measured the stimulus-angle-dependencies of walking direction and turn angle in response to shorter air currents (50 and 100-ms durations). Plots of walking direction versus stimulus angle in response to shorter stimuli also showed linear relationships described by a positive-slope line close to 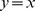 (Figure 3A). The dependency of walking direction on stimulus angle was not significantly affected by reducing the stimulus duration (Table S2). These results suggest that crickets can control their walking direction even if the stimulus finishes before they start walking.

In contrast, the turn angle was smaller in response to shorter stimuli (50, 100 ms) compared with a longer stimulus (200 ms) (Figure 3B). For example, crickets exhibited a small turn in response to shorter (50 and 100 ms) stimuli applied from the side (90° or −90°). Regarding AIC values, a single model containing the effect of stimulus duration was selected over individual models for various durations (Table S3). These results indicate that the stimulus-angle dependency of the turn angle was affected by the stimulus duration and suggest that control of the turn angle requires a longer stimulus duration than control of the walking direction. It is also possible that control of the turn angle may depend on whether the cricket starts to walk before or after termination of the stimulus. To clarify this, we classified responses to a 100-ms stimulus into two categories depending on whether the cricket started walking after the stimulus was terminated (dark blue plots in Figure 3C) or before the stimulus was terminated (light blue plots in Figure 3C). However, there was no difference in dependency of the turn angle on stimulus angle (Figure 3C, Table S3), indicating that control of the turn angle was unaffected by starting to walk before or after stimulus termination, and was only affected by stimulus duration. The mean walking distances of the initial responses were 42.52, 28.21, and 29.07 mm for 200, 100, and 50-ms stimulus durations, respectively (Figure 3D), and the walking distances for the shorter stimuli (50, 100 ms) were significantly shorter than that for the longer stimulus (200 ms) (Table S4), demonstrating that the walking distance was also affected by the stimulus duration.

### Effects of nerve-cord ablation on wind-elicited walking behavior

Ascending signals containing sensory information about air-current direction are conveyed to the cephalic and thoracic ganglia by projection interneurons, including GIs. The motor outputs for walking behavior are delivered by central pattern generators (CPGs) located within the thoracic ganglia [35], [36]. We proposed two types of neural pathways involved in stimulus-angle-dependent control of walking behavior. First, the motor plan of oriented locomotion may be programmed within the brain, based on ascending signals containing the stimulus-angle information, and the command signal descending from the brain controls the walking direction and turn angle. Second, sensory information about the stimulus angle may be decoded from ascending signals in the thoracic ganglia, and the motor outputs regulated directly by local circuits within the thoracic ganglia, based on the decoded information. We tested these hypotheses by partial ablation of the ventral nerve cords containing the ascending and descending axons.

**Figure 4:**
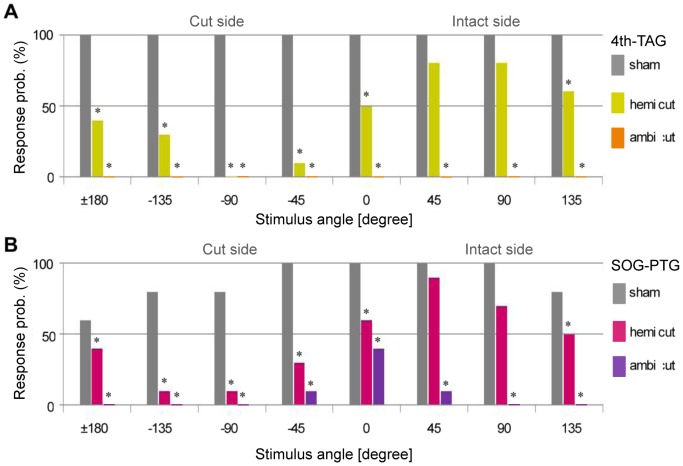
Response probability of wind-elicited walking in various patterns of nerve-cord ablation. Bars represent percentages of animals exhibiting walking behavior in response to air-current stimulation from eight different angles. In the hemi-cut experiments, the angles on the cut side of the nerve cord are indicated as minus values. Control animals (N = 5, for each condition) were subjected to similar dissections to expose the nerve cords, but without ablation. * indicates that the AIC value of the model containing the ablation effect was smaller than that of the model without the ablation effect. A, Response probabilities in 4th-TAG hemi-cut (N = 10, yellow bars) and ambi-cut (N = 10, orange bars) conditions. The probability of response to a stimulus from the cut side was decreased in hemi-cut conditions. Crickets in ambi-cut conditions never responded to air-currents from any angle. B, Response probabilities in SOG-PTG hemi-cut (N = 10, magenta bars) and ambi-cut animals. The response probability to a stimulus from the cut side was decreased in hemi-cut conditions, while a few ambi-cut animals responded to stimulation from behind.

We deleted part or all of the ascending signal from the TAG by cutting one (hemi-cut) or both (ambi-cut) sides of the ventral nerve cords between the 4th AG and TAG. In the 4th-TAG hemi-cut condition, crickets walked in response to air-current stimulation, but the probability of response to anterior and lateral stimuli on the ablated side was decreased (Figure 4A). In the 4th-TAG ambi-cut condition, where the ascending signals were completely lost, air-current stimuli failed to elicit walking behavior. These results indicate that ascending signals from the TAG induce wind-elicited walking behavior, and that even half a side of ascending signals can still trigger wind-elicited walking. We then ablated one or both sides of the nerve cords between the subesophageal and prothoracic ganglia to test the role of descending signals from the cephalic ganglia. In the SOG-PTG hemi-cut condition, crickets responded to an air-puff stimulus, and the response probability in terms of stimulus angle was similar to that in the 4th-TAG hemi-cut condition (Figure 4B). In the SOG-PTG ambi-cut condition, where descending signals were completely lost, crickets rarely responded to stimuli from behind. This suggests that descending signals from the brain were not indispensable for triggering wind-elicited walking.

**Figure 5:**
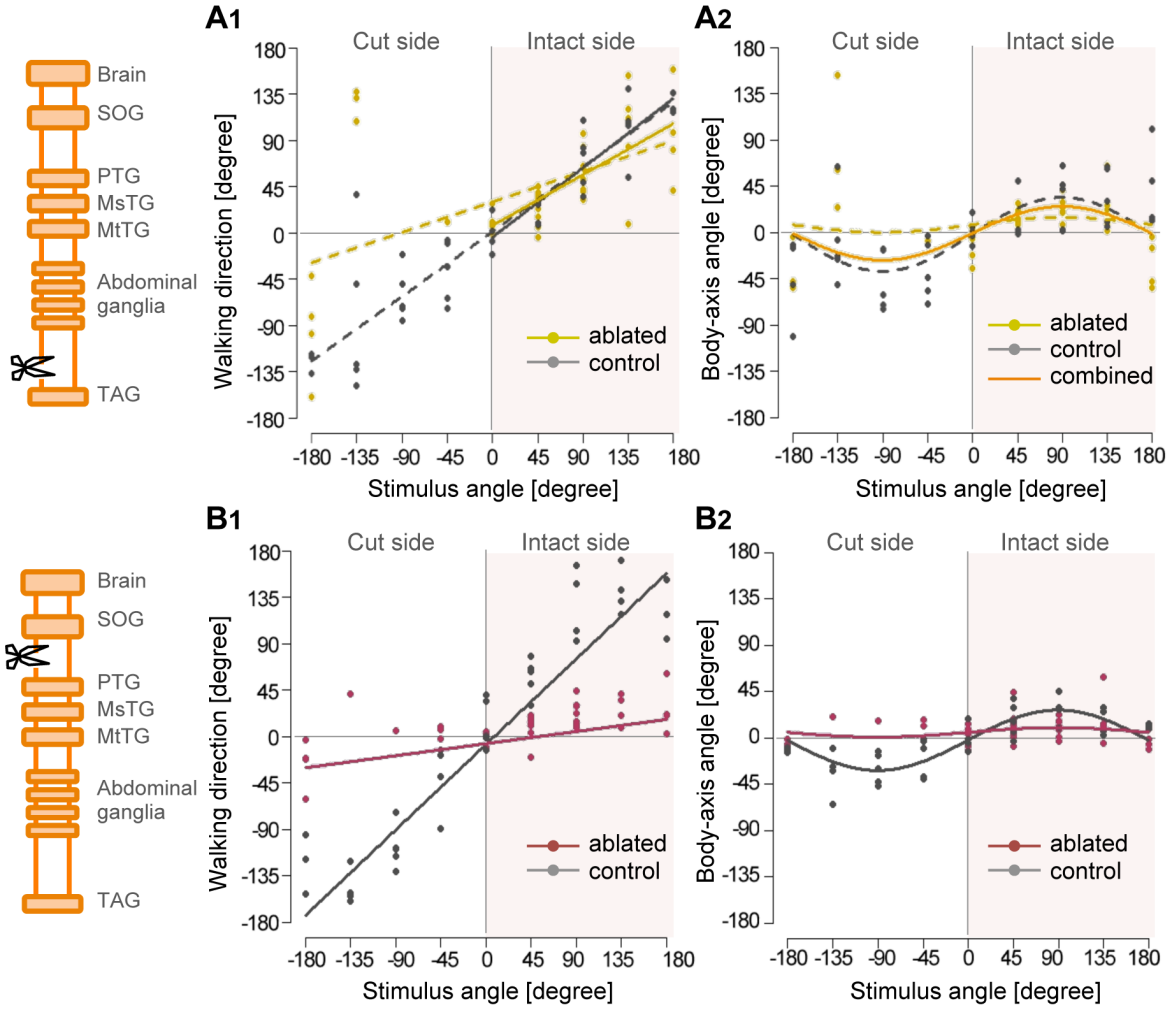
Effects of unilateral nerve-cord ablation on stimulus-angle dependencies of walking direction and turn angle. Left diagrams are diagrammatic representations of the cricket central nervous system, with the ablation site indicated by a scissor mark. A, Plots of walking direction (A1) and turn angle (A2) against stimulus angle in 4th-TAG hemi-cut conditions. Yellow dots and lines represent ablated sample (N = 10), and gray ones represent sham-operated controls (N = 5). Dashed lines in A1 were estimated by individual models for the data in response to stimuli from eight different directions, expressed as 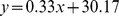 (ablated) and 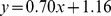 (control). Solid lines in A1 were estimated by models for the data in response to stimuli from five directions on the intact side, expressed as 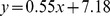 (ablated) and 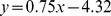 (control). In responses to stimuli from the intact side, stimulus-angle-dependency of walking direction was not significantly affected by nerve-cord ablation between 4th AG and TAG. Distribution of turn angles in A2 was approximated by 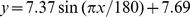 (yellow dashed line for ablated samples) and 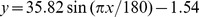 (gray dashed line for controls). Orange solid line represented by 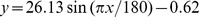 was estimated by the model for all data in ablation and control preparations. B, Plots of walking direction (B1) and turn angle (B2) against stimulus angle in SOG-PTG hemi-cut condition. Magenta dots and lines represent ablation sample (N = 10), and gray ones represent controls (N = 5). The approximate lines in B1 were expressed by 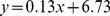 (ablated) and 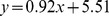 (control). Distributions of turn angles in B2 were approximated by 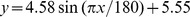 (ablated) and 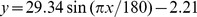 (control).

**Figure 6:**
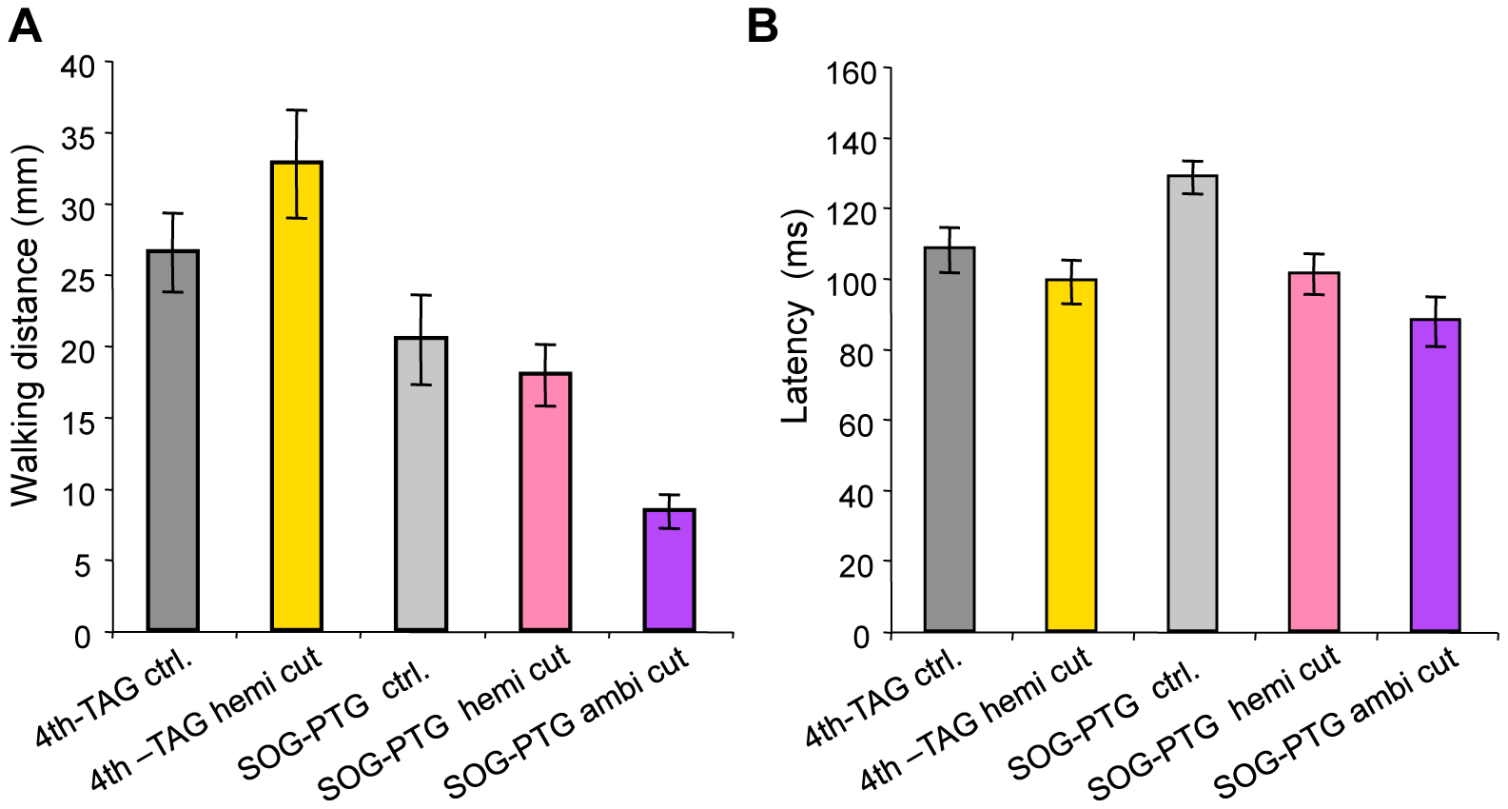
Effects of nerve-cord ablation on walking distance and response latency. Columns and error bars indicate mean ± S.E.M. of all data for each experimental condition. A, Walking distance in animals with various patterns of nerve-cord ablation. Bilateral ablation (ambi-cut) of connective nerve cord between SOG and PTG reduced walking distance. B, Response latency in animals with various patterns of nerve-cord ablation. Fourth AG and TAG hemi-cut had no effects, while hemi- and ambi-cut of the nerve cord at SOG-PTG reduced response latency.

We also analyzed the stimulus-angle-dependencies of walking direction and turn angle during the initial response in nerve-cord ablated animals. In the 4th-TAG hemi-cut condition, the relationship between walking direction and stimulus angle differed from that in control, sham-operated animals (Figure 5A1). Regarding responses to stimuli from the intact side, however, the dependency of walking direction displayed on stimulus angle was similar to that in controls (solid yellow line in Figure 5A1), i.e., crickets walked in the opposite direction to the air-current stimulus on the intact side. Half-side loss of the ascending signals had no significant effect on the stimulus-angle dependency of walking direction on the intact side (Table S2). Turn angle was approximated by the statistical model (GLM, see Materials and Methods) containing no effect of experimental conditions (Figure 5A2, Table S3). Plots of the turn angle were similar to those of controls, such that crickets turned maximally in response to lateral stimulation. These results suggest that crickets can control their walking behavior depending on the stimulus angle, even if half the ascending signals are lost. In contrast, in SOG-PTG ambi- and hemi-cut conditions, crickets were only able to walk in a straight line and did not turn in response to stimulus angle (Figure 5B). Statistical analysis demonstrated that directional control was completely lost if only half of the descending signals were absent (Table S2, S3), indicating that descending signals from the cephalic ganglia were essential for stimulus-angle-dependent control of wind-elicited walking behavior. The mean walking distances were 26.57 and 32.78 mm in the 4th-TAG control and hemi-cut groups, respectively, and 20.49, 17.99, and 8.41 mm in the SOG-PTG control, hemi-cut and ambi-cut groups, respectively (Figure 6A). There were no differences in walking distances between the 4th-TAG and SOG-PTG hemi-cut conditions and their respective controls (Table S4). However, the walking distance in the SOG-PTG ambi-cut animals was much shorter than in controls, suggesting that crickets were only able to walk a few steps when the neural connection between the cephalic and thoracic ganglia was completely interrupted. The mean response latencies were 108.52 and 99.48 ms in the 4th-TAG control and hemi-cut animals, and 129.02, 101.49 and 88.23 ms in the SOG-PTG control, hemi-cut and ambi-cut animals, respectively (Figure 6B). The response latencies in SOG-PTG cut animals were significantly shorter than in controls (Table S5). This result suggests that descending signals may have a delayed effect on wind-elicited walking behavior.

### Effects of laser ablation of single GIs on wind-elicited walking behavior

The functional significance of descending signals for stimulus-angle-dependent control of wind-elicited walking behavior implies that the sensory information about the stimulus angle is conveyed by ascending projection interneurons from the TAG to the cephalic ganglia. The identified GIs are the most likely candidate projection interneurons conveying this information, because of their thick, long axons projecting to the brain ganglion [27]. We examined the influence of laser ablation of single GIs on locomotory parameters in wind-elicited walking behavior. We tested the ability of our laser-beam system to ablate GI function using intracellular recording in an isolated preparation, and checked experimental parameters such as irradiation time and the effective cellular region in terms of functional ablation. Laser irradiation for 900 sec focusing on the initial segment, which is a cellular zone from the dendrites to axon, induced disappearance of the action potential and depolarizing drift of the resting potential (Figure S3). Based on these preparatory experiments, we confirmed that 15 min irradiation was sufficient for GI ablation.

**Figure 7:**
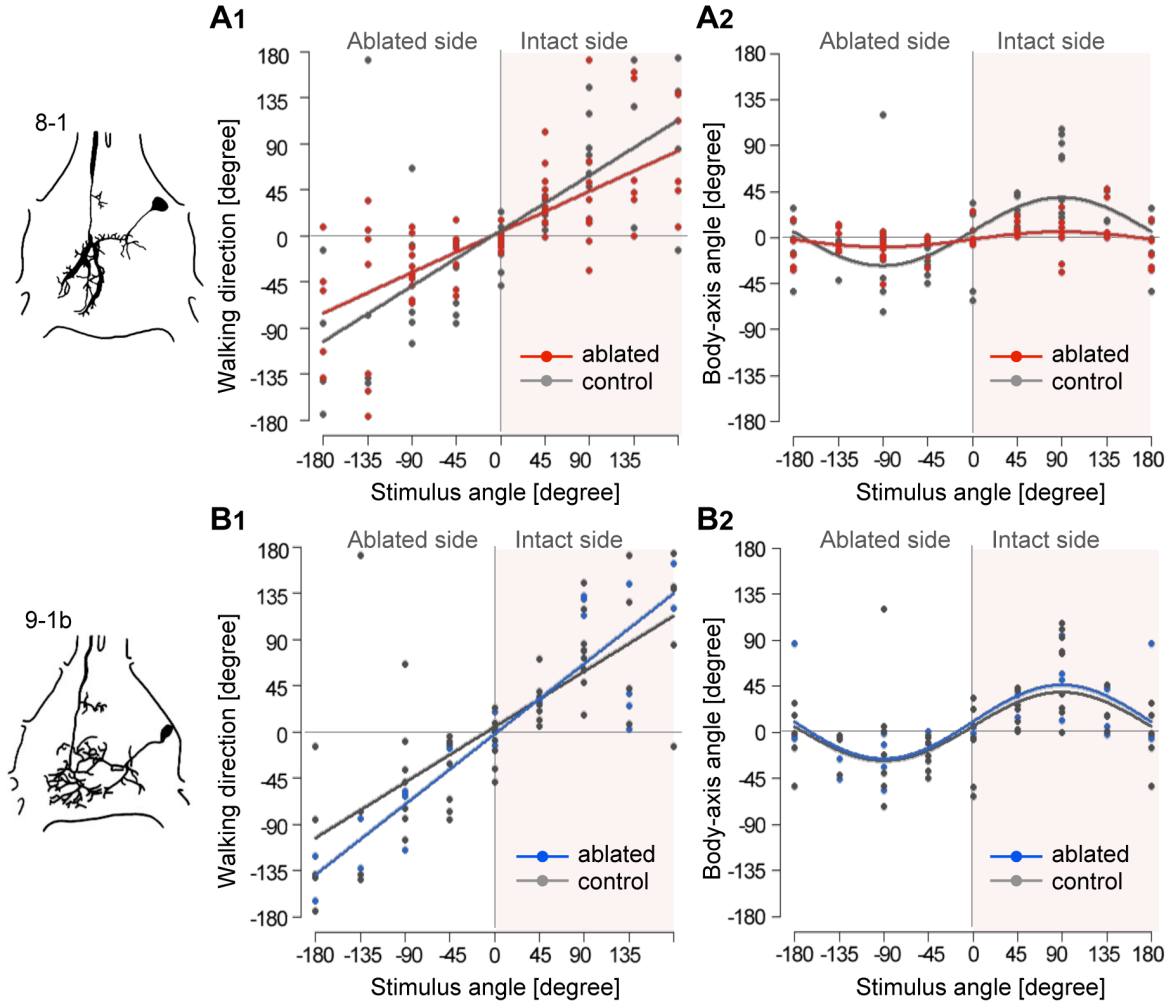
Effects of single-GI ablation on stimulus-angle dependencies of walking direction and turn angle. Left drawings show morphology of GI8-1 (upper) and GI9-1b within TAG. A, Plots of walking direction (A1) and turn angle (A2) against stimulus angle in GI8-1-ablated crickets. Red dots and lines represent ablated sample (N = 6) and gray ones represent controls (N = 8). The approximate lines are given by 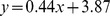 (ablated) and 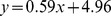 (control). Distributions of the turn angles in A2 were given by 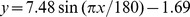 (ablated) and 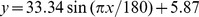 (control). Ablation of GI8-1 had no effect on stimulus-angle dependency of walking direction, but did affect turn angle. Plots of walking direction (B1) and turn angle (B2) against stimulus angle in GI9-1b-ablated crickets. Blue dots and lines represent ablated sample (N = 3) and gray ones represent controls. The approximate lines were given by 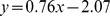 (ablated) and 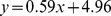 (control). Distributions of the turn angles were approximated by 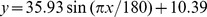 (ablated) and 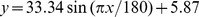 (control). The stimulus-angle dependencies of walking direction and turn angle were not significantly affected by GI9-1b ablation.

We successfully ablated various types of GIs *in vivo*, including GI8-1 (N = 6), 9-1a (N = 1), 9-1b (N = 3), 10-2 (N = 1) and 10-3 (N = 2), and analyzed the results of GI8-1 and GI9-1b ablation in this study. GI8-1 ablation caused no difference in response probability of walking behaviors compared with controls, except in terms of the response to a lateral stimulus to the ablation side (Figure S4). Walking direction was similarly dependent on stimulus angle in GI8-1-ablated and sham-operated controls (Figure 7A1, Table S2). The turn angle in ablated animals, however, was significantly smaller than in control animals (Figure 7A2, Table S3). These results suggest that GI8-1 plays an important role in controlling the turn angle. In the case of GI9-1b ablation, however, walking direction and turn angle depended on the stimulus angle in a similar manner to controls (Figure 7B). There was no significant effect of GI9-1b ablation on stimulus-angle dependencies of walking direction and turn angle (Table S2, S3).

**Figure 8:**
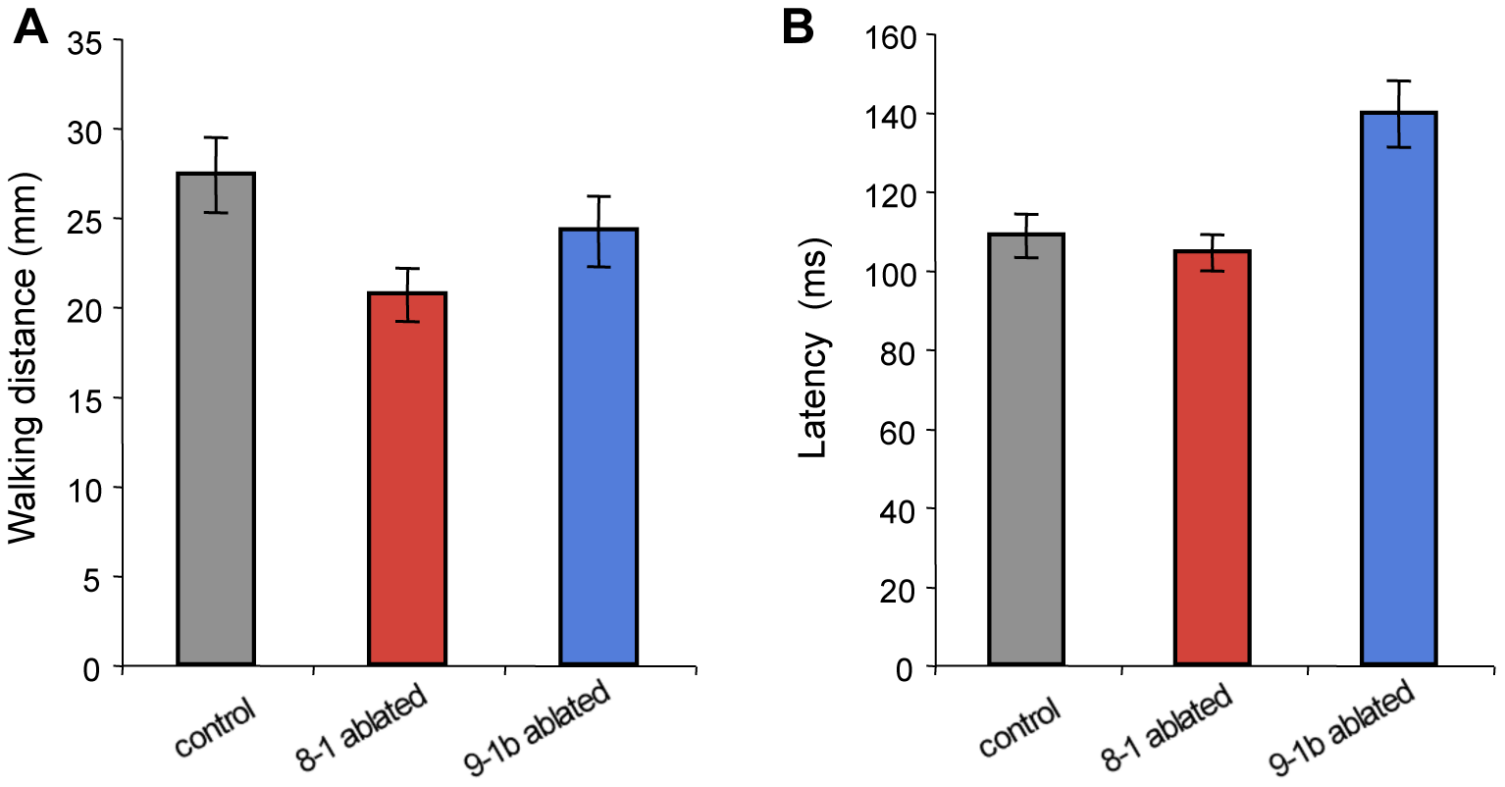
Effects of ablation of single GI on walking distance and response latency. Columns and error bars indicate mean ± S.E.M. of all data for GI8-1-ablated (N = 6), GI9-1b-ablated (N = 3) and control samples (N = 8). A, Walking distances during initial responses in GI8-1- and GI9-1b-ablated crickets. GI8-1 ablation reduced the walking distance, while GI9-1b ablation had no effect on this parameter. B, Response latency in GI8-1- and GI9-1b-ablated crickets. Response latency was prolonged by GI9-1b ablation, but was not significantly affected by GI8-1 ablation.

Laser ablations of GI8-1 and GI9-1b had different effects on other locomotory parameters independent of the stimulus angle. Walking distance of the initial response was significantly shorter in GI8-1-ablated animals (mean 20.69 mm) compared with controls (27.38 mm), though there was no difference between GI9-1b-ablated (24.29 mm) and control animals (Figure 8A, Table S4). Furthermore, there was no difference in response latency between GI8-1-ablated (104.58 ms) and control (108.85 ms) animals, while GI9-1b ablation increased the latency of the walking response (139.77 ms) compared with controls (Figure 8B, Table S5). These results suggest that GI8-1 is involved in the maintenance of walking rather than release of walking, while GI9-1b plays a role in deciding the start timing of walking behavior.

## Discussion

### Stimulus-angle-dependent control in initial response of wind-elicited walking

We used a spherical-treadmill system with high temporal resolution to analyze stimulus-angle-dependent motor control of wind-elicited walking behavior in crickets. Using this system, the directed walking in the x-y plane and turning their body axis were detected simultaneously. The turn angle, however, described a sine-wave-like distribution when plotted against the stimulus angle, while the walking direction bore a linear relationship to the stimulus angle. This indicates that crickets turn their heads to the walking direction when they receive an air-current stimulus from behind or from the side. If the stimulus comes from the front, the cricket darts backwards without any large turn. These different dependencies of both locomotory parameters on stimulus angle were also observed in free-moving crickets, confirming that tethering the cricket to the treadmill had no influence on their walking performance in terms of directional control. Crickets step away from a stimulus source, but do not always face in the opposite direction, suggesting that they prioritize avoidance of the stimulus over turning to the opposite direction to the stimulus source. In cockroaches, turning occurs first and is followed by running away from the stimulus source [8], though our high-speed monitoring of the initial response to air-current stimulation demonstrated that crickets perform their walking and turning movements simultaneously. Furthermore, shorter stimuli were associated with smaller displacements of the body-axis. The effects of stimulus duration on turn-angle control may result from turbulence state of the air-current stimulus at the position of the cerci. The longer duration stimulus would be more laminar and contain more consistent information about stimulus angle. Therefore, it is possible that turn-angle control requires more accurate information about the stimulus angle. In addition to different dependencies of walking direction and turn angle on stimulus angle, this result also suggests that these locomotory parameters may be regulated by distinct neural mechanisms.

### Neural pathway for directional control

To clarify the pathway responsible for neural processing in directional control, we focused on the contribution of neuronal signals descending from the cephalic ganglia. Motor outputs for walking behavior are delivered by CPGs located in the thoracic ganglia [35], [36]. The GIs conveying sensory information about the stimulus angle arborize their axons not only in the thoracic ganglia but also in the cephalic ganglia [27]. We proposed alternative hypotheses: first, that the ascending signals could directly trigger walking behavior within the thoracic ganglia, and second, that accurate control of walking direction and/or turn angle could be executed by descending signals from the cephalic ganglia. Ablation of both sides of the ventral nerve cords between SOG and PTG resulted in a few crickets stepping in response to an air current from behind, thus supporting the idea that afferent signals are directly able to release walking motor outputs in the thoracic ganglia. However, the walking distance in SOG-PTG ambi-cut animals was significantly shorter than that in controls, and crickets with no neural connection between SOG and PTG were unable to walk a long distance steadily compared with intact crickets. This suggests that descending signals are likely to be important for stability and maintenance of walking behavior. Although SOG-PTG hemi-cut crickets walked for a relatively long distance in response to stimulation from the intact side, stimulus-angle-dependent control of the walking direction and turn angle was completely lost. In contrast, the stimulus-angle-dependencies of walking direction and turn angle were maintained on the intact side in 4th-TAG hemi-cut animals, despite deletion of one side of ascending signals as in SOG-PTG hemi-cut animals. This suggests that descending signals from cephalic ganglia are responsible for stimulus-angle-dependent control of wind-elicited walking behavior in crickets. Further, the significant impact of loss of half the descending signals implies that directional control of locomotion relies on bilateral descending signals.

Interestingly, ablation of the nerve cords between the SOG and PTG reduced response latency. Air-current stimulation often induces behaviors other than walking in the cricket, such as jumping, withdrawal, kicking, head stands, abdominal lifting, and hind-leg lifting [10]–[12], [37], [38]. A recent review by Casas and Dangles [13] noted that an air current applied to the cerci elicited at least 14 distinct responses, including evasion, flight, offensive reactions, scanning, freezing, and various reactions during male stridulation. Cricket could thus select the appropriate behavior depending on the stimulus characteristics. Descending signals may suppress initiation of motor activity of thoracic ganglia until the neural processing of decision making for the behavioral choice has been completed within the cephalic ganglia. Nerve-cord ablation between the SOG and PTG may skip this processing and thus reduce response latency.

### Distinct roles of giant interneurons in motor control of wind-elicited walking

The response properties of most GIs have been well investigated, but the role of the information conveyed by each GI in neural processing within the cephalic and thoracic ganglia remains unclear. We examined the roles of each GI by selective ablation *in vivo*. Laser ablation is widely used to disrupt specific cells in neuroscientific studies [39]–[42]. In the cricket cercal system, laser ablation of a single dendritic branch of GI has been used to determine the response properties of specific dendrites to air-current stimulation [43]. The phototoxic fluorescent dye, lucifer yellow CH, or 6-carboxyfluorescein has been used for laser ablation [44], [45]. In this study, we used 6-carboxyfluorescein to avoid damage to the surrounding tissues as a result of laser irradiation, because the excitation wavelength for 6-carboxyfluorescein is longer than that for lucifer yellow.

We successfully ablated five types of GIs, and analyzed the walking behavior in GI8-1- and GI9-1b-ablated crickets. Ablation of GI8-1 disrupted control of the turn angle but not the walking direction, suggesting that the information conveyed by GI8-1 contributes to control of the turn angle. Lesion of specific GIs has also been suggested to affect the initial turn response to air-current stimulation in cockroaches [46]. GI8-1 demonstrates directional sensitivity to its axonal side in terms of its spike number [47]. It is therefore possible that GI8-1 provides directional information on an air-current stimulus from the lateral side of cricket. To rotate their body axis, crickets need to swing their outside legs more than their inside legs, relative to the stimulus, to achieve a turning movement [48]. GI8-1 may be involved in descending control to implement the difference in stride lengths between the left and right legs. Although the walking distance in GI8-1-ablated crickets was shorter than in controls, there was no difference in response latency between them. It is possible that GI8-1 may be also involved in maintenance of the walking behavior.

In contrast, GI9-1b ablation had no discernible effect on stimulus-angle-dependent control in terms of walking direction or turn angle. Although the response properties of GI9-1b have not been clarified in *G. bimaculatus*, GI9-1b revealed little selectivity to direction in its response to air-current stimulation in *A. domestica* [24], [49]. Interestingly, ablation of GI9-1b delayed the start of walking. These results suggest that GI 9-1b plays a key role in determining the onset time of the walking response, but is not involved in stimulus-angle-dependent control or maintenance of wind-elicited walking. In locusts, the identified visual interneuron known as the descending contralateral movement detector (DCMD) is responsible for timely execution of the escape jump in collision-avoidance behavior [45]. Firing of the DCMD neuron does not directly cause the escape behavior, but determines the precise timing of the start of the escape jump. These characteristics of DCMD neurons are similar to those of GI9-1b in the cricket, and are related to the response latency.

GI8-1 has been regarded as a trigger neuron in walking behavior because of the broad sensitivity of its threshold to stimulus direction [50]. In our experiments, however, GI8-1 ablation had no effect on response probability or response latency indicating that GI8-1 is not responsible for triggering the escape behavior. Current injection into GI9-1a, which is a ventral GI (axon running in ventral region of nerve cord) like GI8-1 and GI9-1b, caused turning behavior during walking [29], while current injection into dorsal GIs (axon running in dorsal region of the nerve cord) such as GI9-2, 9-3, 10-2, and 10-3 caused leg movements in tethered crickets [27]. In accordance with our results, previous studies reported that excitation of ventral GIs modulated locomotion, and that dorsal GIs directly evoked motor outputs for leg movements. Ventral GIs may provide sensory information to neural circuits in the cephalic ganglia, which is responsible for directional control or decision making in wind-elicited behaviors. In addition, GI8-1 and GI9-1b appear to play different roles in modulating walking behavior: ascending signals conveyed by GI8-1 maintain walking, while GI9-1b provides important information regulating the timing of walking. The ascending signals involved in the motor control of wind-elicited walking may be divided according to individual types of GIs.

As mentioned in the Introduction, stereotypical escape behaviors are mediated by a few identified neurons, such as the lateral and medial giant fibers in crayfish and Mauthner cells in anamniotes. Their responses are strongly correlated with motor activity, by directly activating the motor neurons responsible for the escape behavior. The directional sensitivity and frequency-tuning property of GIs in the cricket cercal system, however, suggest that these projection interneurons encode various types of stimulus information. In this study, we demonstrated the roles of GI8-1 and GI9-1b in wind-elicited walking behavior. The fact that individual GIs convey distinct types of information to the cephalic and thoracic ganglia suggests the existence of a division of labor within the ascending pathway involved in the cercal sensory system. Additional investigations of other GIs and local ablation of GI axons at the SOG-PTG connection will further our understanding of the functions of individual GIs. More studies are also needed to examine the stimulus-angle-dependency of descending neurons and to clarify the information conveyed by these descending neurons. A full understanding of the behavioral functions of ascending and descending neurons will allow us to describe a global image of the neural network responsible for controlling oriented locomotion.

## Supporting Information

Figure S1 **Statistical analysis using GLM to test the effect of interaction between stimulus angle and condition in artificial data.** The conditions include ‘condition A’ (red), in which the walking direction was linearly correlated with the stimulus angle, and ‘condition B’ (blue), in which the walking direction was constant with the stimulus angle. Solid lines indicate the walking direction of each condition estimated by the model (2)-I. This model containing an effect of interaction between stimulus angle and condition can estimate different correlations between walking direction and stimulus angle for each condition. The AIC value for this model was 102.4. Dashed lines represent walking direction estimated by the model (2)-II. This model did not contain an effect of interaction between stimulus angle and condition and could not estimate differences in correlations between walking direction and stimulus angle for each condition. The AIC of this model was 204.95. Comparing both models in terms of AIC, model (2)-I provided a better estimation of distribution of walking direction against stimulus angle. This result indicates the difference in stimulus-angle dependencies of walking directions between ‘condition A’ and ‘condition B’.
(TIF)

Figure S2
**Stimulus-angle dependencies of walking direction and turn angle in free-moving animals.** The colors of dots and lines represent data acquired in different conditions: green  =  free-moving animals (N = 5) and gray  =  animals tethered on treadmill (N = 10, same data set shown in Figure 2). A, Plots of walking direction against stimulus angle. The approximated lines were given by 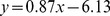 (free-moving) and 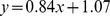 (tethered). B, Distributions of turn angles were approximated by the expressions 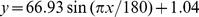 (free moving) and 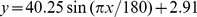 (tethered).(TIF)

Figure S3 **Effect of laser ablation on electrical activity of GIs.** A, Confocal image of GI9-3. Glass microelectrode for intracellular recording was inserted into a neurite close to the axon. White square indicates irradiated region magnified in lower right image. Lower left drawing shows morphology of GI9-3 within TAG. B, Membrane-potential responses of GI9-3 to electrical stimulation of the cercal afferent nerve. Each trace is aligned to stimulus timing, represented as a dashed line. Elapsed time of laser irradiation is indicated on the left of the traces, and the resting potential at that time is indicated above each trace.
(TIF)

Figure S4 **Response probability of wind-elicited walking in GI-ablated animals.** Bars show percentages of animals exhibiting walking behavior in response to air-current stimulation from eight different angles. Stimulus angles on the axonal side of the ablated GIs are indicated as minus values. In control experiments (N = 8, gray bars), the electrode was inserted into the TAG without dye-loading, and the ganglion was irradiated with a blue laser beam. Red bars show the results in GI8-1-ablated animals (N = 6) and blue bars show the results in GI9-1b-ablated animals (N = 3). * indicates that the AIC value of the model containing the ablation effect was smaller than that of the model without the ablation effect.
(TIF)

Table S1 **AIC values of models for stimulus-angle dependencies of locomotory parameters.** All data were measured during the initial response to air-puff stimulation of 200-ms duration of an intact cricket tethered to the treadmill. Models with smaller AIC values, indicated with bold characters, were selected. Walking direction, turn angle and walking distance depended on the stimulus angle, but response latency was independent.
(DOCX)

Table S2 **Statistical analysis of the effects of experimental procedures on stimulus-angle dependency of walking distance.** Center column indicates AIC value of model (2)-I containing the effect of experimental conditions (shown in left column), and right column indicates model (2)-II not containing the condition effects. Shorter stimulus duration (100 ms) and hemi-ablation of the connective nerve cord affected the relationship between the stimulus angle and walking direction. However, the effect of stimulus duration was likely to be small because the AIC values of model (2)-I are close to those of model (2)-II. The effect of 4th-TAG hemi-cut was restricted to the response to stimuli applied from the ablation side.
(DOCX)

Table S3 **Statistical analysis of effects of experimental procedures on stimulus-angle dependency of turn angle.** Center column indicates sum of AIC values of individual models for separate sets of data in experimental conditions (shown in left column), and right column indicates AIC value of single model for combined data in both conditions. Turn angle in response to a stimulus applied from the side was reduced by tethering, shorter stimulus duration, SOG-PTG hemi-cut and ablation of GI8-1.
(DOCX)

Table S4 **Statistical analysis of effects of experimental procedures on walking distance in the initial response.** Center column indicates AIC value of model (4)-I containing the effect of experimental conditions (shown in left column), and right column indicates model (4)-II not containing the condition effects. Walking distance was reduced by shorter stimulus duration, 4th-TAG hemi-cut, SOG-PTG ambi-cut and ablation of GI8-1.
(DOCX)

Table S5 **Statistical analysis of effects of experimental procedures on response latency.** Center column indicates AIC value of model (4)-I containing the effect of experimental conditions (shown in left column), and right column indicates model (4)-II not containing the condition effects. Ablation of connective nerve cord between SOG and PTG reduced the latency, while ablation of GI9-1b delayed the start of walking.
(DOCX)
