# Supplementary figures and images for: Neural Basis of Stimulus-Angle-Dependent Motor Control of Wind-Elicited Walking Behavior in the Cricket Gryllus bimaculatus
Source: PLoS One. 2013 Nov 14;8(11):e80184. doi: 10.1371/journal.pone.0080184 (PMC3828193; doi:10.1371/journal.pone.0080184)

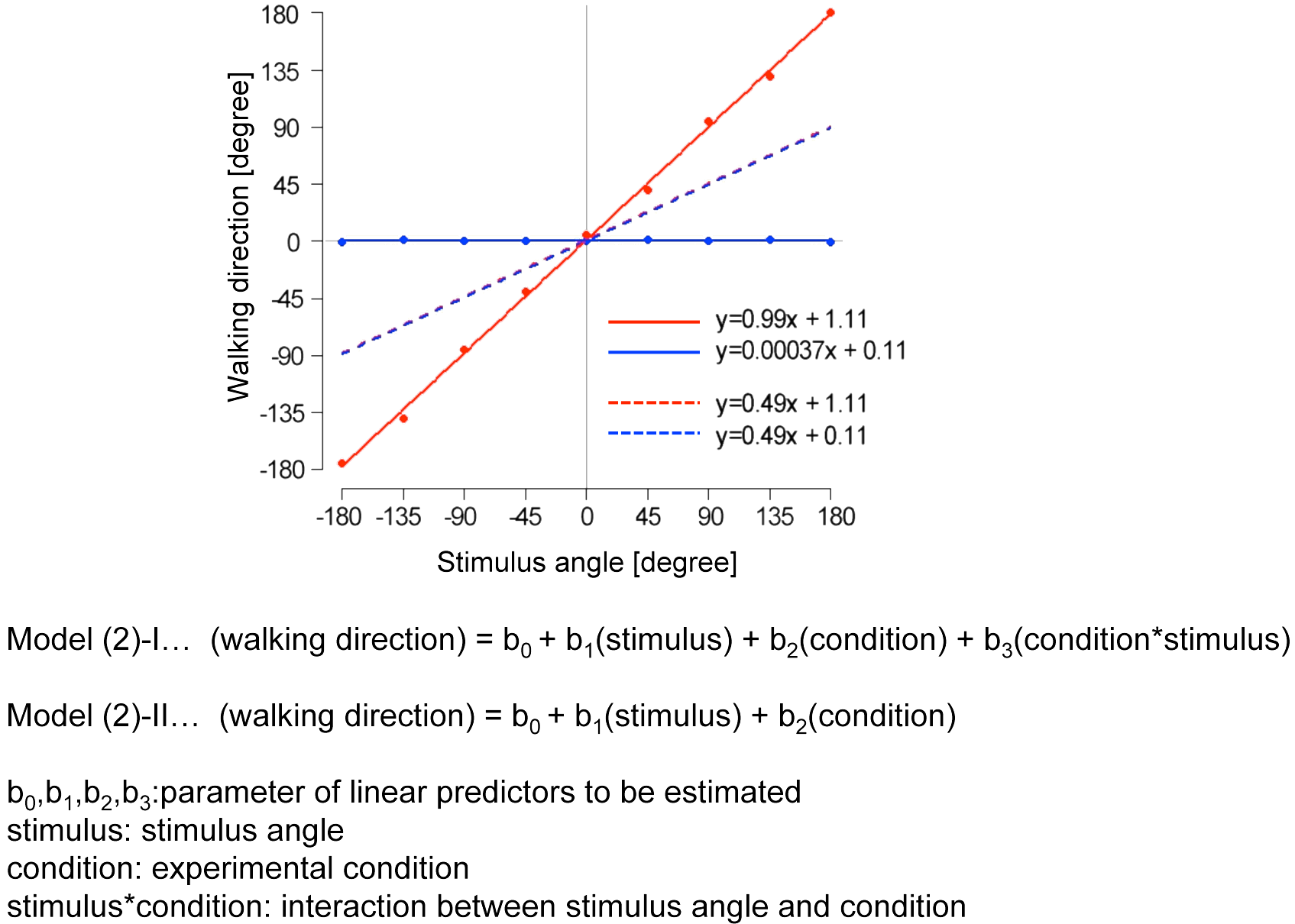

Supplement: Figure S1 — Statistical analysis using GLM to test the effect of interaction between stimulus angle and condition in artificial data. The conditions include ‘condition A’ (red), in which the walking direction was linearly correlated with the stimulus angle, and ‘condition B’ (blue), in which the walking direction was constant with the stimulus angle. Solid lines indicate the walking direction of each condition estimated by the model (2)-I. This model containing an effect of interaction between stimulus angle and condition can estimate different correlations between walking direction and stimulus angle for each condition. The AIC value for this model was 102.4. Dashed lines represent walking direction estimated by the model (2)-II. This model did not contain an effect of interaction between stimulus angle and condition and could not estimate differences in correlations between walking direction and stimulus angle for each condition. The AIC of this model was 204.95. Comparing both models in terms of AIC, model (2)-I provided a better estimation of distribution of walking direction against stimulus angle. This result indicates the difference in stimulus-angle dependencies of walking directions between ‘condition A’ and ‘condition B’. (TIF) [file pone.0080184.s001.tif]

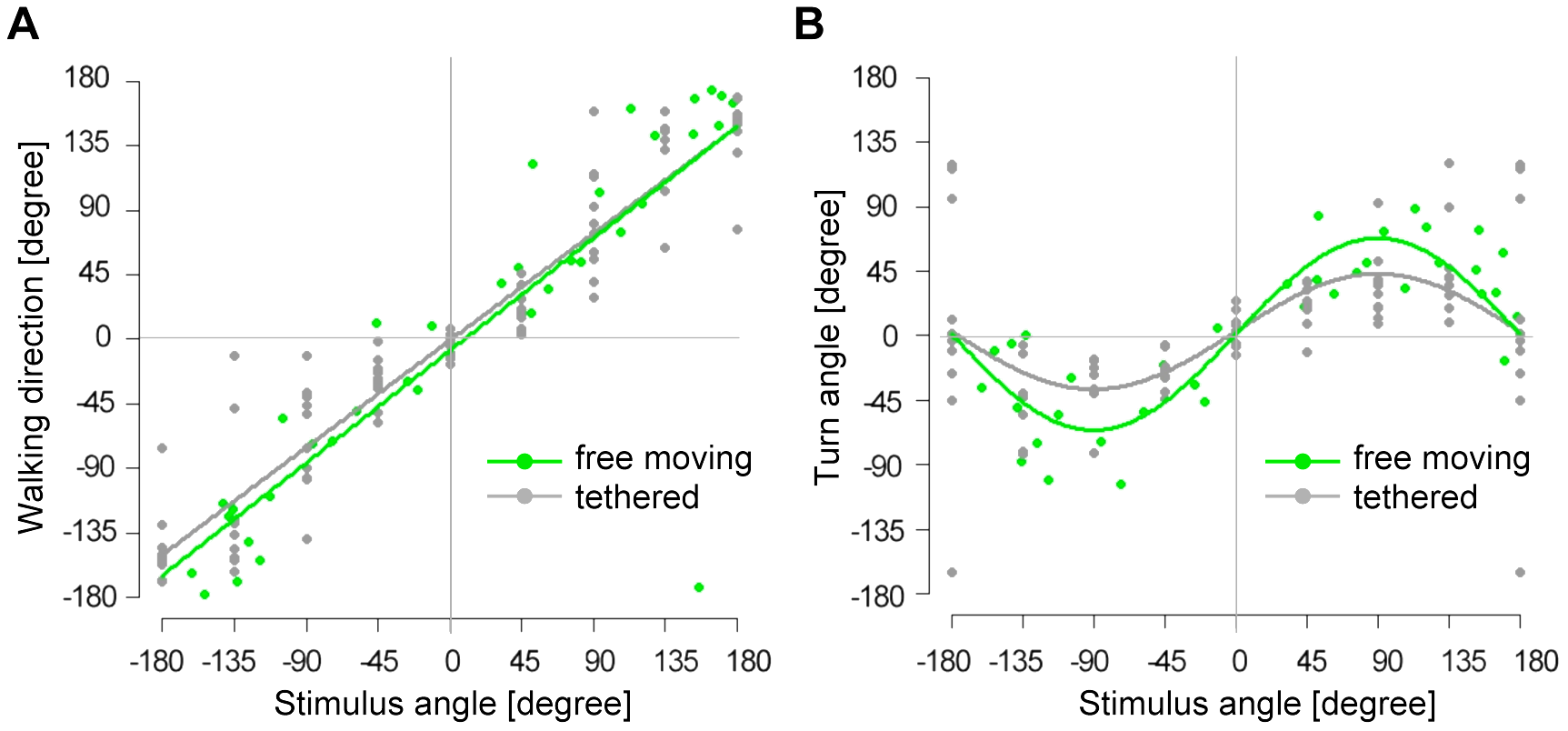

Supplement: Figure S2 — Stimulus-angle dependencies of walking direction and turn angle in free-moving animals. The colors of dots and lines represent data acquired in different conditions: green = free-moving animals (N = 5) and gray = animals tethered on treadmill (N = 10, same data set shown in Figure 2). A, Plots of walking direction against stimulus angle. The approximated lines were given by (free-moving) and (tethered). B, Distributions of turn angles were approximated by the expressions (free moving) and (tethered). (TIF) [file pone.0080184.s002.tif]

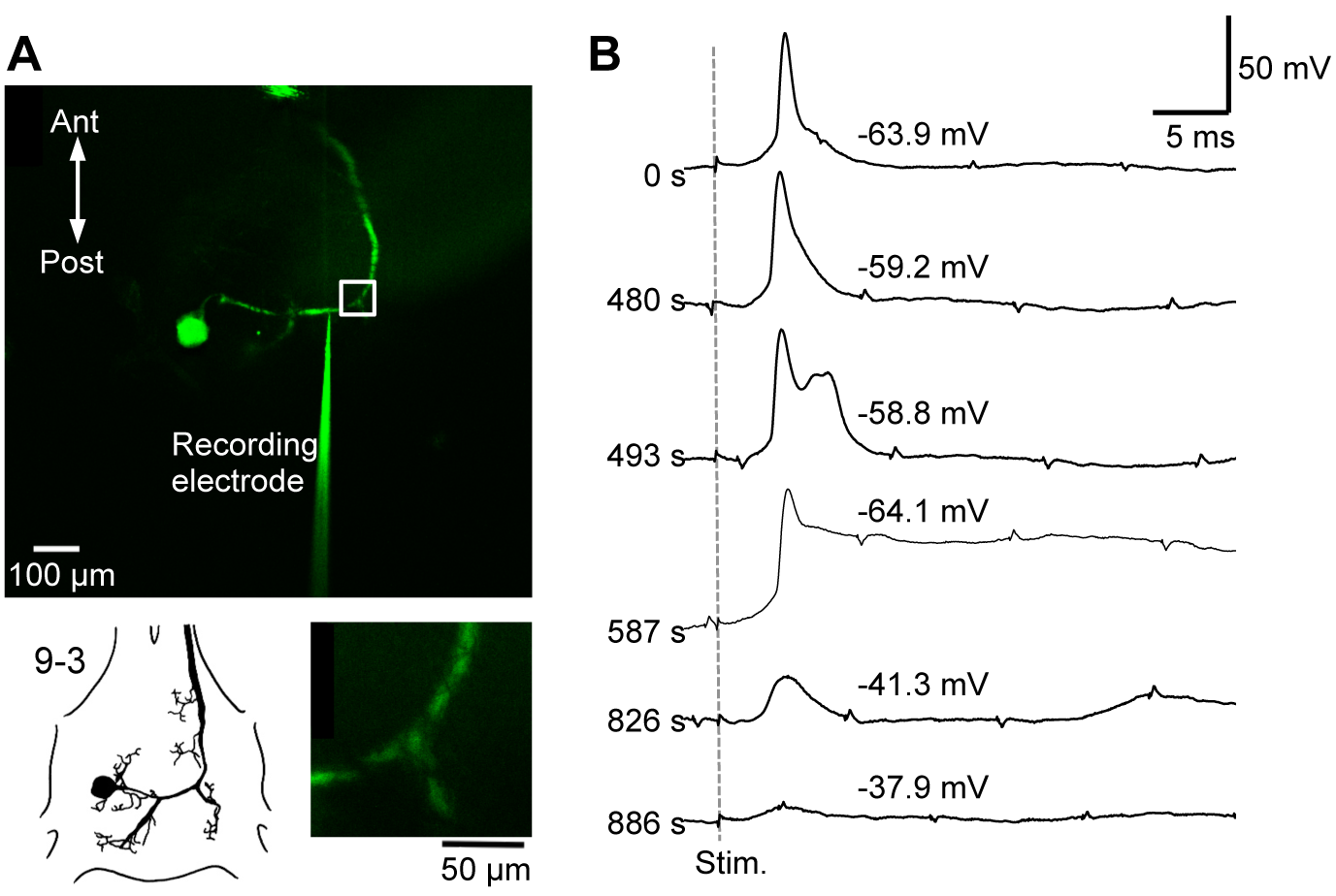

Supplement: Figure S3 — Effect of laser ablation on electrical activity of GIs. A, Confocal image of GI9-3. Glass microelectrode for intracellular recording was inserted into a neurite close to the axon. White square indicates irradiated region magnified in lower right image. Lower left drawing shows morphology of GI9-3 within TAG. B, Membrane-potential responses of GI9-3 to electrical stimulation of the cercal afferent nerve. Each trace is aligned to stimulus timing, represented as a dashed line. Elapsed time of laser irradiation is indicated on the left of the traces, and the resting potential at that time is indicated above each trace. (TIF) [file pone.0080184.s003.tif]

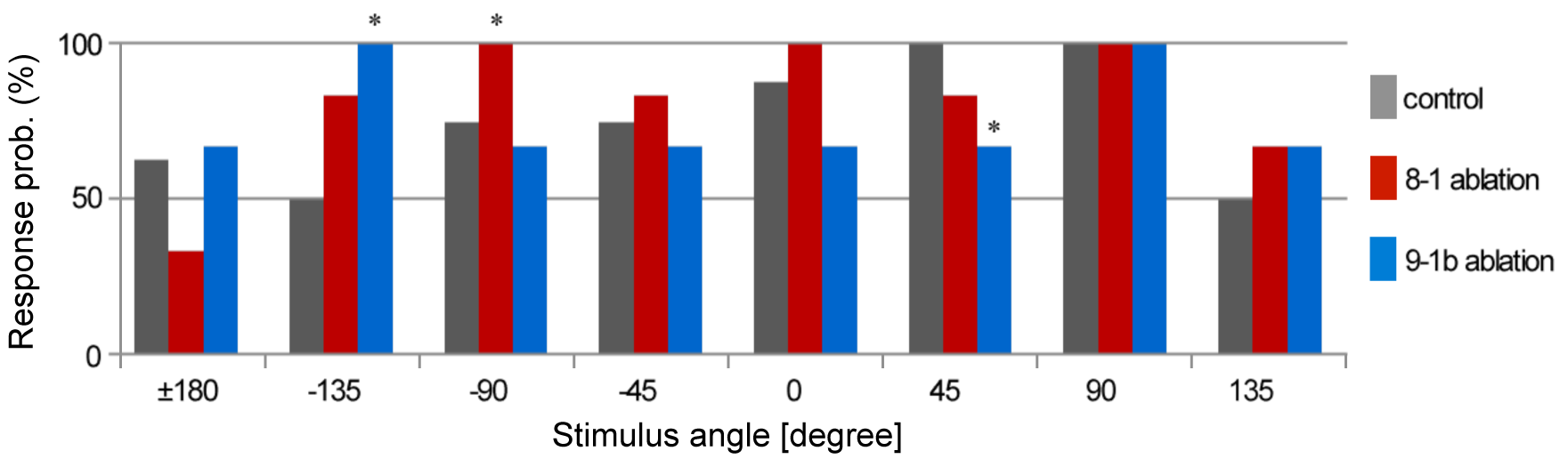

Supplement: Figure S4 — Response probability of wind-elicited walking in GI-ablated animals. Bars show percentages of animals exhibiting walking behavior in response to air-current stimulation from eight different angles. Stimulus angles on the axonal side of the ablated GIs are indicated as minus values. In control experiments (N = 8, gray bars), the electrode was inserted into the TAG without dye-loading, and the ganglion was irradiated with a blue laser beam. Red bars show the results in GI8-1-ablated animals (N = 6) and blue bars show the results in GI9-1b-ablated animals (N = 3). * indicates that the AIC value of the model containing the ablation effect was smaller than that of the model without the ablation effect. (TIF) [file pone.0080184.s004.tif]
