# Supplementary material for: Neural Basis of Stimulus-Angle-Dependent Motor Control of Wind-Elicited Walking Behavior in the Cricket Gryllus bimaculatus
Source: PLoS One. 2013 Nov 14;8(11):e80184. doi: 10.1371/journal.pone.0080184 (PMC3828193; doi:10.1371/journal.pone.0080184)
Supplement: Table S1 — AIC values of models for stimulus-angle dependencies of locomotory parameters. All data were measured during the initial response to air-puff stimulation of 200-ms duration of an intact cricket tethered to the treadmill. Models with smaller AIC values, indicated with bold characters, were selected. Walking direction, turn angle and walking distance depended on the stimulus angle, but response latency was independent. (DOCX) [file pone.0080184.s005.docx]

| walking direction | | turn angle | | walking distance | | Response latency | |
| --- | --- | --- | --- | --- | --- | --- | --- |
| model (1)-I | model (1)-II | model (3)-I | model (3)-II | model (4)-I | model (3)-II | model (4)-I | model (4)-II |
| **856.68** | 1066.5 | **921.79** | 947.55 | **810.37** | 814.71 | 846.85 | **845.02** |
