# Supplementary material for: Neural Basis of Stimulus-Angle-Dependent Motor Control of Wind-Elicited Walking Behavior in the Cricket Gryllus bimaculatus
Source: PLoS One. 2013 Nov 14;8(11):e80184. doi: 10.1371/journal.pone.0080184 (PMC3828193; doi:10.1371/journal.pone.0080184)
Supplement: Table S2 — Statistical analysis of the effects of experimental procedures on stimulus-angle dependency of walking distance. Center column indicates AIC value of model (2)-I containing the effect of experimental conditions (shown in left column), and right column indicates model (2)-II not containing the condition effects. Shorter stimulus duration (100 ms) and hemi-ablation of the connective nerve cord affected the relationship between the stimulus angle and walking direction. However, the effect of stimulus duration was likely to be small because the AIC values of model (2)-I are close to those of model (2)-II. The effect of 4th-TAG hemi-cut was restricted to the response to stimuli applied from the ablation side. (DOCX) [file pone.0080184.s006.docx]

| condition | model (2)-I | model (1)-II |
| --- | --- | --- |
| free moving vs tethered | 1281 | **1279.4** |
| 100 ms vs 200 ms | **1055.4** | 1055.6 |
| 50 ms vs 200 ms | 1292.6 | **1291.5** |
| 4th-TAG hemi-cut vs control (both sides) | **882.07** | 898.82 |
| 4th-TAG hemi-cut vs control (intact side) | 533 | **532.66** |
| SOG-PTG hemi-cut vs control | **744.36** | 836.14 |
| 8-1 ablated vs control | 1414.5 | **1414.2** |
| 9-1b ablated vs control | 918.86 | **917.12** |
