# Supplementary material for: Neural Basis of Stimulus-Angle-Dependent Motor Control of Wind-Elicited Walking Behavior in the Cricket Gryllus bimaculatus
Source: PLoS One. 2013 Nov 14;8(11):e80184. doi: 10.1371/journal.pone.0080184 (PMC3828193; doi:10.1371/journal.pone.0080184)
Supplement: Table S3 — Statistical analysis of effects of experimental procedures on stimulus-angle dependency of turn angle. Center column indicates sum of AIC values of individual models for separate sets of data in experimental conditions (shown in left column), and right column indicates AIC value of single model for combined data in both conditions. Turn angle in response to a stimulus applied from the side was reduced by tethering, shorter stimulus duration, SOG-PTG hemi-cut and ablation of GI8-1. (DOCX) [file pone.0080184.s007.docx]

| condition | SUM of individual models | single model for combined data |
| --- | --- | --- |
| free moving vs tethered | **1263.06** | 1275.43 |
| 100 ms vs 200 ms | **1577.08** | 1595.27 |
| 50 ms vs 200 ms | **1275.69** | 1321.09 |
| before stim. off vs after stm. off  (100-ms duration) | 660.03 | **655.28** |
| 4th-TAG hemi-cut vs control | 849.32 | **848** |
| SOG-PTG hemi-cut vs control | **647.84** | 662.38 |
| 8-1 ablated vs control | **1195.06** | 1252.41 |
| 9-1b ablated vs control | 844.50 | **840.35** |
