# Supplementary material for: Neural Basis of Stimulus-Angle-Dependent Motor Control of Wind-Elicited Walking Behavior in the Cricket Gryllus bimaculatus
Source: PLoS One. 2013 Nov 14;8(11):e80184. doi: 10.1371/journal.pone.0080184 (PMC3828193; doi:10.1371/journal.pone.0080184)
Supplement: Table S4 — Statistical analysis of effects of experimental procedures on walking distance in the initial response. Center column indicates AIC value of model (4)-I containing the effect of experimental conditions (shown in left column), and right column indicates model (4)-II not containing the condition effects. Walking distance was reduced by shorter stimulus duration, 4th-TAG hemi-cut, SOG-PTG ambi-cut and ablation of GI8-1. (DOCX) [file pone.0080184.s008.docx]

| condition | model (4)-I | model (4)-II |
| --- | --- | --- |
| 100 ms vs 200 ms | **1402.4** | 1414.6 |
| 50 ms vs 200 ms | **1180.8** | 1186.8 |
| 4th-TAG hemi-cut vs control | **708.18** | 708.61 |
| SOG-PTG hemi-cut vs control | 600.66 | **599.46** |
| SOG-PTG ambi-cut vs control | **329.13** | 335.98 |
| 8-1 ablated vs control | **1037.1** | 1042.8 |
| 9-1b ablated vs control | 680.2 | **679.28** |
