# Supplementary material for: Neural Basis of Stimulus-Angle-Dependent Motor Control of Wind-Elicited Walking Behavior in the Cricket Gryllus bimaculatus
Source: PLoS One. 2013 Nov 14;8(11):e80184. doi: 10.1371/journal.pone.0080184 (PMC3828193; doi:10.1371/journal.pone.0080184)
Supplement: Table S5 — Statistical analysis of effects of experimental procedures on response latency. Center column indicates AIC value of model (4)-I containing the effect of experimental conditions (shown in left column), and right column indicates model (4)-II not containing the condition effects. Ablation of connective nerve cord between SOG and PTG reduced the latency, while ablation of GI9-1b delayed the start of walking. (DOCX) [file pone.0080184.s009.docx]

| condition | model (4)-I | model (4)-II |
| --- | --- | --- |
| 4th-TAG hemi-cut vs control | 855 | **853.66** |
| SOG-PTG hemi-cut vs control | **769.17** | 779.86 |
| SOG-PTG ambi-cut vs control | **420.69** | 430.83 |
| 8-1 ablated vs control | 1343.3 | **1341.3** |
| 9-1b ablated vs control | **867.8** | 875.33 |
